# Excitonic Structure and Dynamics in Two-Dimensional Halide Perovskites

**DOI:** 10.1021/acs.nanolett.6c02134

**Published:** 2026-07-27

**Authors:** Fabian Lie, Linn Leppert

**Affiliations:** † MESA+ Institute for Nanotechnology, 3230 University of Twente, 7500 AE Enschede, The Netherlands; ‡ School of Metallurgy and Materials, University of Birmingham, B15 2SE Birmingham, United Kingdom

**Keywords:** two-dimensional halide perovskites, exciton fine structure, exciton−phonon coupling, spectroscopy, first-principles calculations

## Abstract

Layered two-dimensional halide perovskites (2D HPs) have emerged as a versatile platform for studying strongly bound excitons in the presence of spin–orbit coupling, structural symmetry breaking, and pronounced coupling to lattice vibrations. In this Mini-Review, we focus on the monolayer limit of this class of materials and highlight recent progress in understanding excitonic fine structure and optical selection rules, chiral and spin-dependent optical response, exciton–phonon coupling and exciton relaxation pathways, and the breakdown of simple band-edge pictures in 2D HPs in which organic and inorganic electronic states hybridize or more complex band structures emerge as a consequence of metal-cation chemistry. Throughout, we emphasize the role of atomistic theoretical and computational frameworks in interpreting the rapidly growing experimental literature. Together, these developments highlight 2D HPs as an unusually rich class of low-dimensional materials for studying spin-optical and excitonic quantum phenomena.

## Graphical abstract

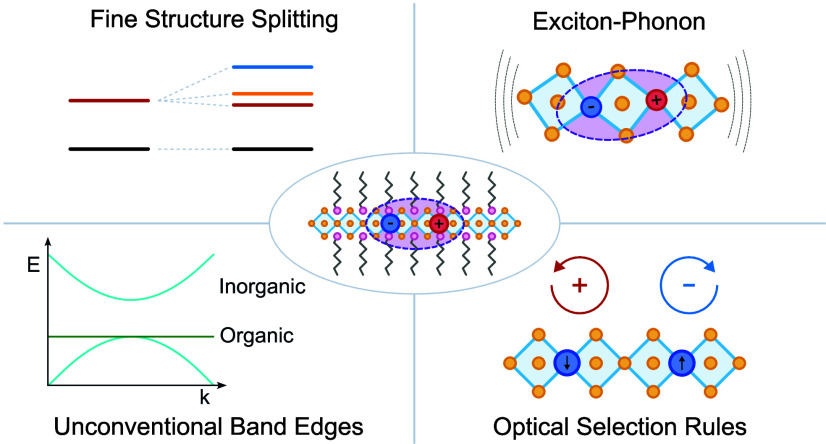

## Introduction

Layered organic–inorganic halide perovskites (HPs) have emerged as a distinctive class of low-dimensional semiconductors in which rich physics arises as a consequence of the coupling of bound electron–hole pairs (excitons), spin and orbital angular momenta and phonons. In the monolayer limit (shown in Figure 1a), these materials consist of atomically defined metal-halide sheets separated by organic spacer layers, creating a natural quantum-well structure with strong quantum and dielectric confinement effects.^1–5^ Excitons in these materials are sensitive probes of symmetry, dielectric environment, structural dynamics, and spin-dependent interactions.

**Figure 1:**
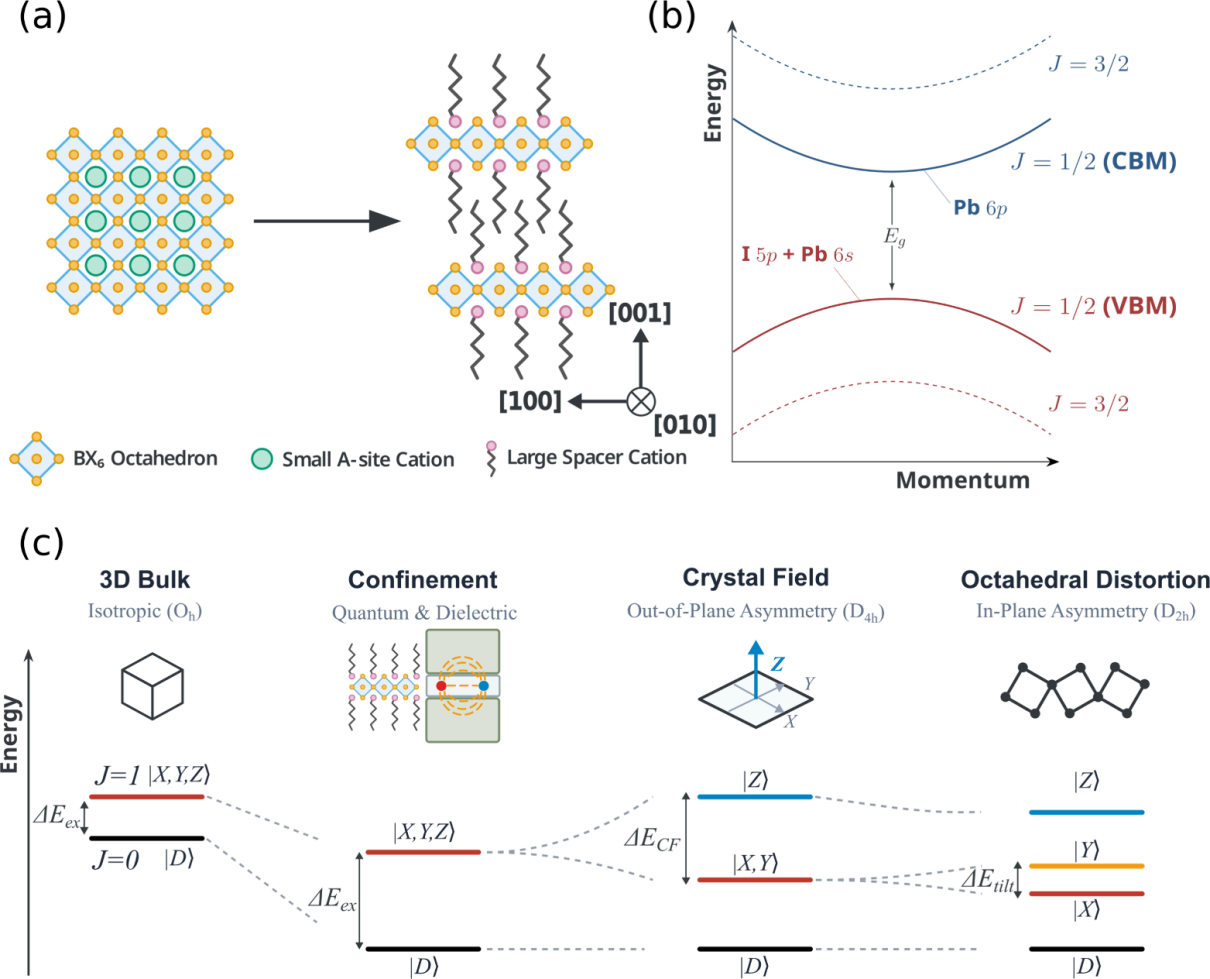
(a) Schematic of a 3D (left) and 2D HP structure with perovskite monolayers separated by organic spacer molecules. (b) Schematic band structure of a typical 2D HP, indicating orbital contributions to the band-edge states and the total angular momenta of these states. (c) Schematic excited-state fine-structure diagrams, highlighting exchange-energy splitting Δ*E*_*ex*_, crystal-field splitting Δ*E*_*CF*_, and distortion-induced energy-level splitting Δ*E*_*tilt*_. The labeled point groups (*O*_*h*_, *D*_4*h*_, *D*_2*h*_) denote successive idealized symmetry-lowering steps rather than the full symmetry of any specific compound. Real 2D HPs, in which interfacial hydrogen bonding and steric effects from the organic spacer induce octahedral tilts and other distortion patterns, correspond to the lower-symmetry (*D*_2*h*_ or below) panel.

This combination places two-dimensional (2D) HPs in the broader landscape of excitonic 2D materials alongside transition metal dichalcogenides, hexagonal boron nitride and other van der Waals systems. Yet their microscopic physics is qualitatively different. In van der Waals semiconductors, covalently bonded inorganic layers are weakly coupled, and excitonic physics can be tuned by precise control of interlayer composition, stacking, and tilting.^6–8^ In contrast, layered perovskites derive their properties from the interplay between a “soft” inorganic perovskite framework and its organic environment that acts simultaneously as dielectric barrier, structural template, and, in some cases, an electronically active component. In conjunction with the compositional flexibility afforded by both the inorganic and the organic layers, this hybrid character gives rise to a level of tunability that is unusual even within the diverse family of 2D materials.

Much of the early interest in 2D HPs centered on their large exciton binding energies, structural and compositional tunability, robust stability and potential for optoelectronic applications. More recently, their rich excitonic physics, and their suitability as a tunable, experimentally accessible platform for studying it, has come into focus. Their low-energy excited states are strongly affected by exchange interactions, crystal-field effects, and strong spin–orbit coupling, leading to a fine structure of dark and bright states with distinct polarization. At the same time, octahedral tilting and other symmetry breaking distortions of the perovskite network, dielectric anisotropy, and coupling with phonons can strongly modify exciton energies, optical selection rules, and excited-state relaxation pathways. These effects have brought 2D HPs in conversation with broader themes in modern condensed-matter and materials physics, including dark-bright exciton conversion, Rashba/Dresselhaus physics, chiral optical response, and exciton–phonon coupling.

In this Mini-Review, we focus on the monolayer limit of 2D HPs, not attempting a comprehensive survey of thickness-dependent trends, compositional tunability, structural diversity and stability. Instead, we approach this field from a theoretical and computational perspective, in close dialogue with the rapidly growing experimental literature. Our aim is to highlight 2D HPs as a platform for studying excitonic fine structure, spin and chirality, their coupling with phonons and structural dynamics, and the breakdown of simple band-edge pictures when organic and inorganic degrees of freedom become strongly coupled. Together, these aspects position 2D HPs as a versatile class of low-dimensional materials that serves as an emerging platform for spin-optical and excitonic quantum phenomena.

## Excitonic Fine Structure in 2D Perovskites

The excitonic fine structure of 2D HPs is one of the defining features of this material class.^9–12^ In contrast to a simple picture of a single optically active exciton at the absorption onset, the lowest excitonic states in this class of materials form a closely spaced manifold whose ordering and splittings are governed by spin–orbit coupling, electron–hole exchange, reduced dielectric screening, and structural symmetry breaking. Particular attention has been devoted to the relative ordering of the dark and bright states, the role of the out-of-plane polarized exciton, and the extent to which confinement, lattice distortions, and dynamical effects affect this manifold.^9,10,13–19^

At the band edge of lead- and tin-based HPs, the relevant electronic states are strongly influenced by the heavy-metal atom. The valence-band maximum (VBM) is derived from halide *p* and metal *s* states, whereas the conduction-band minimum (CBM) has substantial metal *p* character. Strong spin–orbit coupling (SOC), particularly at the conduction-band edge, leads to band-edge states that are described by spin–orbit-coupled total angular momentum rather than pure spin and orbital characters (Figure 1b). As a consequence of valence and conduction band edges with total angular momentum of *J* = 1/2, the excitonic manifold consists of one optically forbidden dark singlet |*D*⟩ (*J* = 0) and three optically allowed bright triplet states (*J* = 1), commonly denoted |*X*⟩, |*Y*⟩, and |*Z*⟩.^11,20–22^ This excited-state ordering is observed in both 3D and 2D HPs and supported by symmetry analysis, first-principles calculations, and magneto-optical experiments.^11,20–24^

The splittings within this manifold are controlled primarily by the electron–hole exchange interaction, schematically shown in the first column of Figure 1c.^25^ Because exchange is short-ranged, it becomes stronger as the electron and hole are confined to the same region of space. In 2D HPs this overlap is enhanced by two related consequences of reduced dimensionality. First, quantum confinement localizes carriers within the 2D inorganic slab. Second, dielectric confinement arises because the organic spacer layers have a much smaller dielectric constant than the inorganic framework, reducing screening and strengthening the Coulomb attraction between electron and hole.^4,5,15^ The same ingredients that produce large exciton binding energies therefore also increase bright-dark splittings, as indicated in the second column of (Figure 1c). Experimentally, bright-dark splittings of roughly 18–30 meV have been reported for several 2D perovskites, substantially larger than in 3D perovskite nanocrystals, and these splittings increase systematically with exciton binding energy, consistent with an exchange-driven origin.^10,19^

Beyond the bright-dark splitting, the internal structure of the bright triplet reflects the reduced symmetry of the layered crystal. In an idealized high-symmetry picture, two bright states are associated with orthogonal in-plane transition dipole moments, while the third state carries an out-of-plane dipole moment and is shifted in energy by crystal-field effects,^23,26^ schematically shown in the third column of Figure 1c. In real materials octahedral tilting, layer asymmetry, strain, and other structural distortions further lower the symmetry and can lift the residual degeneracy of the in-plane states, rotate the preferred polarization axes, and modify the energetic position and optical activity of the out-of-plane state,^18,27–29^ shown in the fourth column of Figure 1c, and discussed in detail below.

Furthermore, depending on the charge of the organic spacer, 2D HPs are commonly classified as Ruddlesden–Popper phases, in which monovalent spacer cations separate the inorganic sheets and adjacent layers are mutually offset, or Dion–Jacobson phases, in which divalent spacer cations bridge neighboring layers and tend to align them. This distinction primarily affects the interlayer spacing and dielectric environment and hence the magnitude of exciton binding and fine-structure splittings. At small interlayer distances, out-of-plane exciton delocalization has been predicted for model Dion–Jacobson phases.^30^ Experimentally synthesized Dion–Jacobson perovskites with small interlayer distances indeed have significant out-of-plane band dispersion indicating interlayer electronic coupling,^31^ but excitonic properties of these materials have not been studied.

Because the organic spacer usually affects the band-edge physics indirectly through its influence on the inorganic framework rather than through direct electronic participation,^32,33^ the excitonic fine structure becomes highly sensitive to lattice geometry, including the magnitude and pattern of in- and out-of-plane octahedral tilts. This sensitivity is one reason why different 2D HPs can display quantitatively different fine-structure splittings.

The current microscopic picture of fine structure has been established through a combination of spectroscopy and theory, which agree on the qualitative ordering of the low-energy manifold. Magneto-photoluminescence and magneto-absorption measurements have been especially important: by mixing bright and dark excitonic states, an applied magnetic field grants optical access to otherwise weakly allowed transitions, and it is through such measurements that the lowest dark exciton was brightened and the bright-dark splittings of 18–30 meV noted above were extracted across several 2D HPs.^10,19^

Polarization-resolved optical measurements help assign the orientation of excitonic transition dipole moments, because in-plane and out-of-plane excitons couple to different components of the electromagnetic field. By matching not only relative energy levels and oscillator strength, but also the transition dipole moment, to theoretical results, a more confident mapping between experimental and computational results can be made.^34^ Polarization-resolved reflectance has placed the out-of-plane |*Z*⟩ state energetically above the split in-plane states,^19^ while polarization-resolved photoluminescence (PL) resolves the in-plane manifold of (PEA)_2_PbI_4_ (PEA = phenethylammonium), which we discuss in the following section.^9,21^

It is worth noting that not every low-energy emission feature reported in 2D HPs should be viewed as part of the intrinsic exciton fine structure. In particular, additional red-shifted PL peaks have in some cases been discussed in terms of charged excitonic complexes (trions),^35^ while in other cases phonon-assisted emission, localized (self-trapped) excitons, or other sample-specific effects have been invoked.^21,36–39^ Finally, time-resolved measurements can provide complementary support for fine-structure assignments by revealing state-specific population dynamics.^40,41^ The associated relaxation pathways are discussed in more detail in the context of exciton–phonon coupling below.

On the theory side, first-principles many-body perturbation theory within the *GW* ^42,43^ and Bethe–Salpeter Equation^44–47^ (BSE) frameworks have been central to establishing the microscopic picture of exciton fine structure in 2D HPs. The earliest *GW*+BSE study on this class of systems, by Molina-Sánchez and co-workers, considered a freestanding Cs_2_PbBr_4_ monolayer and clarified the symmetry of the band-edge states, the large exciton binding energy caused by reduced dimensionality and screening, and the presence of a lowest-energy dark exciton.^12^ Around the same time, Giorgi et al. reported *GW*+BSE calculations for butylammonium lead iodide 2D HPs, showing how excitonic absorption evolves from mono- to bilayer models, although without focusing in detail on the fine-structure splittings themselves.^48^ Subsequent work has demonstrated that the low-energy excitonic manifold in layered perovskites can be considerably more complex. Filip et al., for example, showed that dielectric screening by the organic spacer strongly affects the exciton binding energy and reported for (PMA)_2_PbBr_4_ (PMA = phenylmethylammonium) and Cs_2_PbBr_4_ a manifold of low-energy states arising from transitions between four near-degenerate valence and conduction band states.^15^ More recently, Quarti and co-workers combined *GW*+BSE and symmetry analysis for Cs_2_PbI_4_ and confirmed the characteristic sequence of dark, in-plane, and out-of-plane excitons, while also systematically testing the effect of structural distortions on excitonic fine structure.^18^ The central qualitative predictions of these calculations are confirmed by experiment: the dark state is revealed through magneto-optical brightening,^10,19^ the bright-dark splittings match the exchange-driven magnitudes extracted from the same measurements, and the position of the out-of-plane |*Z*⟩ state above the in-plane doublet is confirmed by polarization-resolved reflectance.^19^ Furthermore, they have been complemented by model-Hamiltonian and effective-mass approaches, which provide physical intuition for the roles of exchange, crystal-field splitting, and symmetry lowering, and offer a convenient framework for analyzing deviations induced by tilting, confinement, and spin splitting.^14,23,24,49–51^

Taken together, these studies have established the essential ingredients governing exciton fine structure, but they also suggest that the connection between calculated excitonic states and the full low-energy spectral structure observed experimentally in specific 2D HP compounds is not yet completely understood. Here it is important to distinguish two roles played by structural distortions. Static, symmetry-lowering distortions are essential ingredients of the fine structure itself since they are direct consequences of the reduced symmetry and will depend sensitively on material composition. By contrast, dynamic disorder, electron– and exciton–phonon coupling, and defects, broaden the relevant transitions and can obscure the underlying excitonic manifold. In practice, the fine structure is therefore resolved at cryogenic temperatures, in high-quality samples, and using field- and polarization-selective techniques. Separating the intrinsic fine structure from extrinsic and dynamical contributions nonetheless remains an ongoing challenge that will require increasingly detailed combined theoretical and experimental analysis.

## Transition Dipole Moments, Optical Selection Rules, and Chirality

Beyond their energetic ordering, the lowest excitons in 2D HPs are also distinguished by how they couple to light. In the long-wavelength optical limit, where the photon momentum is negligible on the scale of the Brillouin zone, absorption and emission predominantly probe direct excitons with nearly zero center-of-mass momentum. Within the electric-dipole approximation, valid when the electromagnetic field is effectively uniform over the spatial extent of the excitation, the oscillator strength of an exciton *S* is determined by the projection of the light polarization vector ê onto its transition dipole moment $\mu_{S}=\left\langle 0\right|\mathrm{r̂}\left|S\right\rangle$ and is proportional to ${\left|ê\cdot \mu_{S}\right|}^{2}$ . The magnitude of **μ**_*S*_ therefore controls the strength of the optical transition, whereas its orientation determines which polarization components of the electromagnetic field can excite or probe a given exciton. The direction and symmetry of **μ**_*S*_ therefore provide an additional and experimentally accessible fingerprint of exciton character.

Polarization-resolved optical measurements have been central to resolving these closely spaced fine-structure components through their anisotropic coupling to polarized light.^9,11,21,52^ (PEA)_2_PbI_4_, which has been studied in particular detail, illustrates both the power of this approach and the interpretive ambiguities that remain. Low-temperature polarization-resolved PL on (PEA)_2_PbI_4_ resolved four spectral features *X*_1_–*X*_4_, with the brightest features *X*_2_ and *X*_3_ each splitting into two orthogonally polarized components (Figure 2a–c).^9^ By their orthogonal in-plane polarization and large oscillator strength, *X*_2_ and *X*_3_ are naturally identified with the in-plane |*X*⟩ and |*Y*⟩ excitons of the idealized fine-structure picture; the doubling of each into two peaks, however, leaves the bright manifold with twice as many in-plane states as predicted, and the origin of these additional features remains debated.

**Figure 2:**
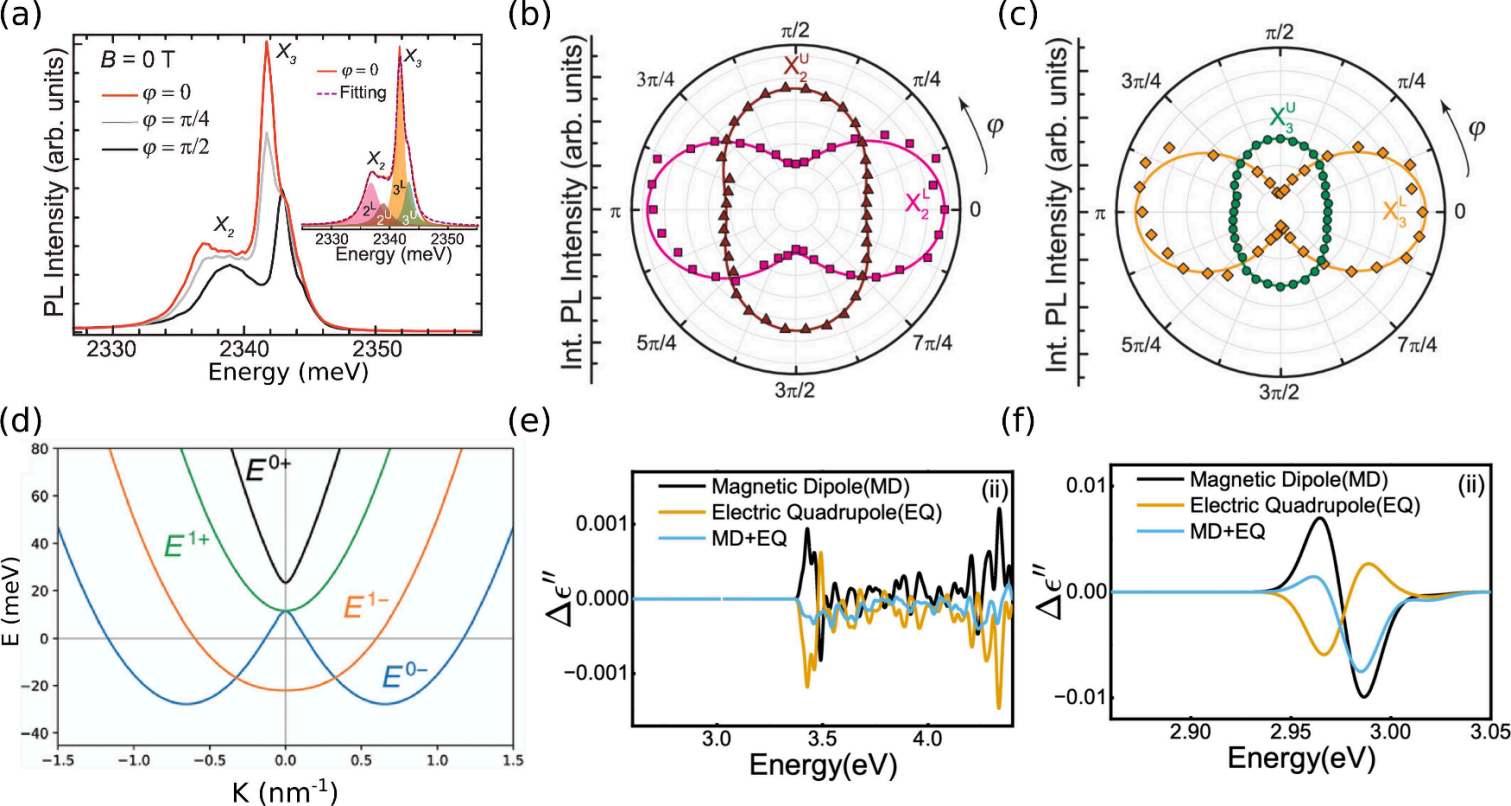
(a) Linearly polarized PL spectra of (PEA)_2_PbI_4_ at 5.9 K and *B* = 0 T for three different in-plane detection angles φ. Inset: Typical fitting to a spectrum with four Lorentzian-shaped components. Reproduced from ref 9. Copyright 2020 American Chemical Society. Integrated PL intensity versus in-plane polarization angle φ for (b) $X_{2}^{L}$ (pink squares), $X_{2}^{U}$ (brown triangles) and (c) $X_{3}^{L}$ (orange diamonds), $X_{3}^{U}$ (green circles). Reproduced from ref 9. Copyright 2020 American Chemical Society. (d) Calculated exciton dispersion for 2D HP including SOC and Rashba-splitting terms. The black line represents the dark, orange the out-of-plane, and blue and green the in-plane excited states. Reproduced from ref 14. Copyright 2021 Royal Society of Chemistry. Calculated circular dichroism of (S-NEA)_2_PbBr_4_, separately showing magnetic dipole (black), electric quadrupole (yellow), and combined (blue) contributions, (e) without and (f) with excitonic effects. Reproduced from ref 53. Available under a CC-BY 4.0 license. Copyright 2025 Xu and Qiu.

Several microscopic interpretations have been advanced. Muñoz-Matutano and co-workers attributed part of the broadening of the *X*_2_/*X*_3_ region to spectral diffusion driven by dynamical charge fluctuations in the organic layer, with sharper four-peak structures recovered after clamping the organic spacers by an interleaved hBN layer.^52^ Posmyk et al. proposed that the additional peaks instead reflect exciton–polaron emission,^21^ building on earlier spectroscopic evidence for vibronic replicas in 2D HPs.^54^ In this picture, the polaronic manifold inherits the selection rules of the underlying bright states and reproduces the observed multiplicity without invoking additional purely electronic states. A complementary structural interpretation, based on combined polarization-resolved spectroscopy and *GW*+BSE calculations, assigns the doubling to two inequivalent perovskite layers in the (PEA)_2_PbI_4_ unit cell, which support two sets of layer-localized intralayer excitons; within the same framework, the previously unassigned *X*_4_ feature is identified as a manifold of interlayer excitons arising from this layer doubling.^34^ The polaronic and structural mechanisms are not mutually exclusive, and their relative contributions to the observed spectra of (PEA)_2_PbI_4_ have not yet been definitively disentangled.

Beyond the in-plane manifold, polarization-resolved reflectance measurements have placed the out-of-plane exciton |*Z*⟩ energetically above the split in-plane states, settling a previously unresolved point.^19^

A distinct route to modified optical selection rules arises from inversion-symmetry breaking. In noncentrosymmetric 2D HPs, strong SOC can generate Rashba- or Dresselhaus-like spin splittings of the band-edge states.^55,56^ In contrast to 3D HPs, where inversion-symmetry breaking is generally constrained to polar, displacive distortions,^57^ layered compounds offer several additional structural routes to such effects, including the intrinsic asymmetry of the organic–inorganic layering, octahedral tilts and rotations,^28^ spacer-induced distortions,^58–60^ and dynamic lattice fluctuations.^61–63^ This richer structural landscape also underlies other consequences of inversion-symmetry breaking in 2D perovskites, including macroscopic polarization and hybrid improper ferroelectricity.^64,65^ In (PEA)_2_PbI_4_, for example, calculations suggest that specific orientations of the organic spacer can locally break inversion symmetry and induce spin-textured band edges in the inorganic framework.^63^

At the single-particle level, inversion-symmetry breaking can give rise to slightly momentum-offset valence- and conduction-band extrema as well as nontrivial spin textures near the band edge, whose details depend on the distortion pattern.^28,66^ For exciton physics, the most important consequences are expected to be changes in the spin character and optical accessibility of the lowest excitonic states and, in some cases, a shift of the minimum of the exciton dispersion to finite center-of-mass momentum **Q**. Model-Hamiltonian work has shown that Rashba spin splitting can, in principle, invert the ordering of bright and dark excitons, depending on the relative signs and magnitudes of the electron and hole Rashba parameters as shown in Figure 2d.^14^ A momentum offset between the CBM and VBM can additionally render the lowest excitonic transition effectively indirect in momentum space, which suppresses direct radiative recombination and can prolong emission lifetimes. This mechanism has been invoked to explain slow recombination in 3D HPs,^67^ although clear experimental confirmation remains lacking. Likewise, both the magnitude and the microscopic relevance of Rashba-like effects in 2D HPs remain debated, particularly in systems that are globally centrosymmetric or in which spectroscopic signatures admit alternative explanations.^68^

An important subset of noncentrosymmetric structures is formed by chiral crystals which lack any improper symmetry that would superimpose them on their mirror image. In chiral 2D HPs, this handedness can be transferred from the organic spacer molecules to the inorganic framework through asymmetric hydrogen-bonding interactions with the halide ions, lowering the crystal symmetry to a chiral space group.^69–75^ The resulting optical hallmark is circular dichroism (CD), i.e., different absorption of left- and right-circularly polarized light.^76–78^ Experimentally, chiral 2D HPs display CD signals of opposite sign for opposite enantiomers at the energies of the excitonic resonances. This is nontrivial because the corresponding band-edge states are derived predominantly from the inorganic framework rather than from electronically active frontier orbitals of the chiral organic spacer, as outlined above. The observation therefore suggests that the excitonic transitions inherit the handedness of the crystal environment through structural chirality transfer to the inorganic lattice and the resulting modification of the excitonic wave functions and selection rules. Microscopically, CD lies beyond the electric-dipole approximation: it arises from interference between electric- and magnetic-dipole transition amplitudes, with electric-quadrupole contributions also entering depending on the formulation.

Theoretical descriptions of this response differ substantially in both level of approximation and physical emphasis. Tight-binding work has shown that circular optical response can already be formulated beyond the electric-dipole approximation through magnetic-dipole and electric-quadrupole matrix elements, albeit without explicit excitonic effects.^77^ More recent first-principles studies based on *GW*+BSE have demonstrated that excitonic effects are essential, with the independent-particle contribution leading to much weaker differential absorption (Figure 2e and f).^53,78^ The earlier of these studies augmented the excitonic transition matrix elements with magnetic-dipole contributions only,^78^ whereas a subsequent full-minimal-coupling formulation avoids the truncated multipole expansion and shows explicitly that both magnetic-dipole and electric-quadrupole contributions can be important.^53^ Complementary effective-mass modeling reaches a related but conceptually distinct conclusion: excitonic CD emerges from parity mixing of the band-edge Bloch functions induced by polar distortions, which makes magnetic-dipole transitions symmetry-allowed, together with level mixing within the excitonic fine structure driven by the interplay of nonchiral Rashba-like and chiral/helical spin-texture terms.^79^

It is worth noting that differential absorption of left- and right-circularly polarized light is not restricted to structurally chiral crystals. Recently, circularly polarized-light absorption was demonstrated in a centrosymmetric crystal, where the effect was traced to interference between linear dichroism and linear birefringence,^80^ i.e., a distinct mechanism with different symmetry requirements and a characteristic sign inversion upon sample flipping. Related cautionary arguments also arise in the context of chiral 2D HPs, where apparent CD can result from morphology or oriented anisotropic structures rather than intrinsic chiral excitonic response.^79^

The coexistence of strong excitonic effects, oriented transition dipoles, and tunable symmetry breaking makes 2D HPs appealing for polarized emitters and chiral photonics,^81–83^ selective spin transport,^84–86^ and, more broadly, cavity-based strong-coupling platforms,^87–89^ although these directions remain far less explored than the fine structure itself.

## Exciton–Phonon Coupling and Relaxation Pathways

Exciton–phonon coupling is a general feature of semiconductor physics, but plays a particularly important role in 2D HPs because strongly bound excitons coexist with soft, polar, anisotropic vibrational degrees of freedom, for which deviations from the harmonic phonon approximation can be significant. As a result, excitonic properties in these materials cannot be understood purely in terms of static electronic structure. Lattice dynamics can renormalize exciton energies, contribute to dielectric screening, and influence how excitons relax, localize, or dissociate after photoexcitation.^90^ To this end, various methods exist that can be used for the detection of electron– and exciton-phonon coupling in 2D HPs as reviewed in ref 91.

A first important aspect in this context is the screening of the electron–hole interaction itself. In 2D HPs, large exciton binding energies reflect the quantum confinement of electrons and holes within the inorganic slab, combined with the dielectric contrast between inorganic and organic sublattices.^15^ Additionally, in polar semiconductors ionic motion can contribute to the effective screening of electronic excitations, thereby renormalizing quasiparticle and excitonic energies.^92^ This effect has been discussed for 3D HPs, where phonon screening can substantially reduce exciton binding energies and modify optical spectra.^93–95^ In 2D HPs, the quantitative role of phonon screening is expected to be more limited due to their large exciton binding energies, although a fully microscopic assessment remains largely missing.

Another question is whether exciton–phonon coupling also influences the stability of excitons at the organic–inorganic interface. Beyond serving as a dielectric boundary, this interface may in some cases provide a pathway for phonon-assisted exciton dissociation or charge separation, particularly in systems where structural fluctuations or more complex band-edge character weaken the picture of a rigidly confined exciton.^96^ This possibility is motivated by experimental evidence that excitons in 2D HPs are strongly dressed by lattice motion and can couple to distinct vibrational coordinates,^40,54,97,98^ while interlayer electronic delocalization and exciton dissociation have both been observed in regimes where the organic and inorganic sublattices are more strongly coupled.^99–101^

One particularly instructive example of phonon-controlled exciton dynamics is the relaxation between bright and dark excitonic states. As detailed above, in many 2D HPs the lowest-energy excitonic state is optically dark.^10,18^ In 2D HPs, strong spin–orbit coupling mixes spin and orbital character, so that phonon-mediated scattering from the bright (*J* = 1) to the dark (*J* = 0) excited state is expected to be efficient for phonons of appropriate symmetry and energy. Experimentally, however, intense PL is often observed even at cryogenic temperatures,^9,22,34^ indicating that relaxation into the dark state is not always efficient. A recently proposed explanation for this apparent contradiction invokes a phonon-bottleneck mechanism for bright-dark relaxation.^102^ In this picture, relaxation between bright and dark excitons requires a phonon mode resonant with the bright-dark splitting. In many semiconductors this condition is easily met, because excitons couple efficiently to acoustic phonons, whose energies make a resonant mode available essentially irrespective of the splitting. For example, in quasi-2D CdSe nanoplatelets, bright-dark relaxation is efficient and only weakly sensitive to the splitting magnitude based on this mechanism.^103^ In 2D HPs, the energies of acoustic phonon modes lie below the dark-bright splitting, and the soft, polar lattice makes Fröhlich coupling to LO phonon modes the dominant exciton–phonon interaction.^104,105^ Since LO phonons are discrete and relatively widely spaced, they are typically not resonant with the bright-dark splitting so that relaxation can proceed only through much rarer higher-order processes. The pathway is thereby bottlenecked, and the bright-state population can remain far above the value expected from thermal equilibrium (Figure 3a and b). By combining microscopic modeling with magneto-optical spectroscopy, ref 102 showed that this mechanism can produce bright-exciton populations several orders of magnitude larger than those predicted from Boltzmann statistics alone. Another proposed explanation for the intense PL is the presence of biexcitonic states. By using coherent nonlinear spectroscopy to determine biexciton binding energies, a second emission peak at low temperatures can be assigned to these biexcitonic states, broadening the signal expected from a purely excitonic picture.^107^

**Figure 3:**
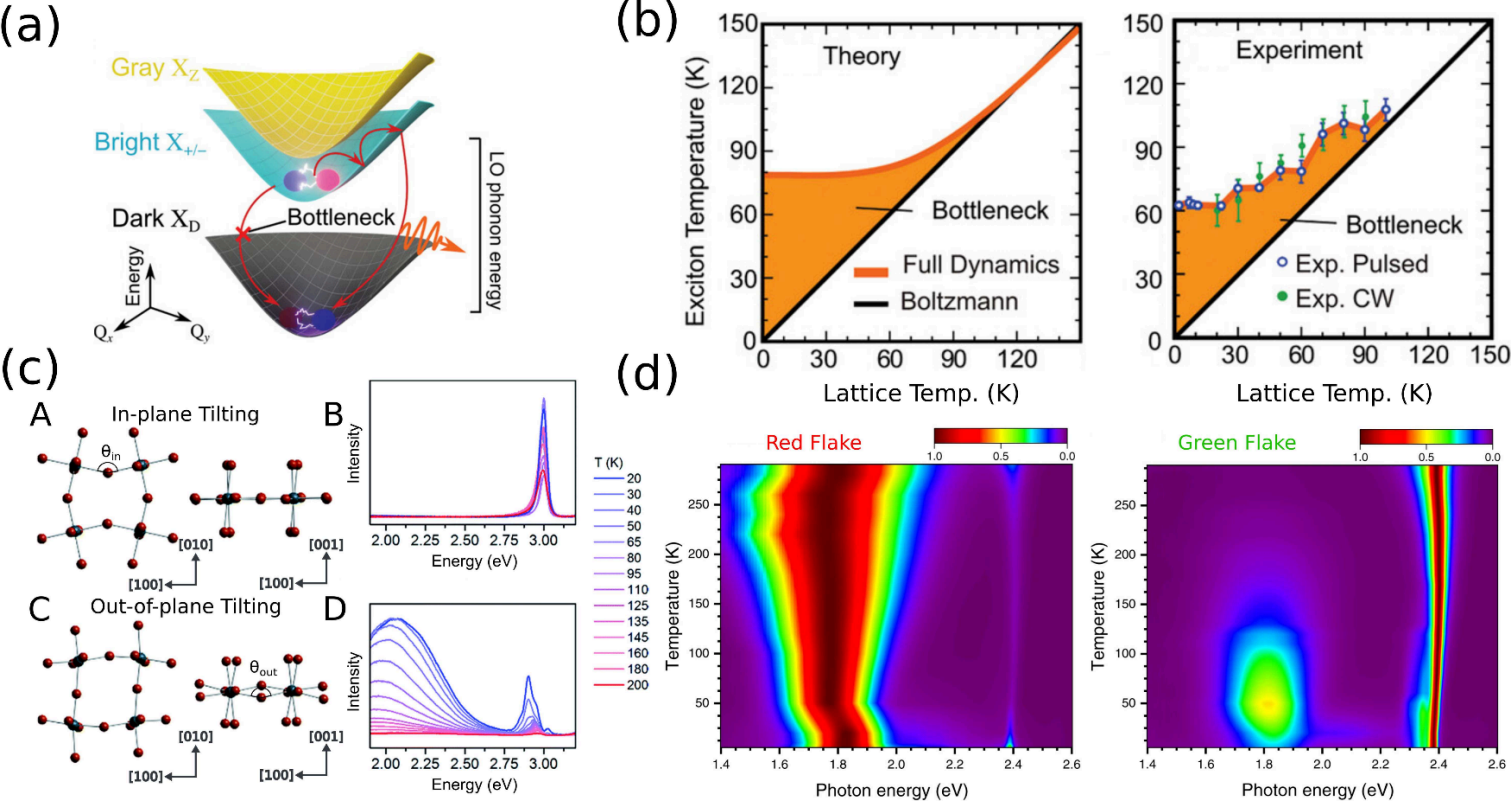
(a) Schematic of exciton fine structure, including bright (X_+_, X_–_), gray (X_*Z*_), and dark exciton states (X_*D*_) and their relaxation dynamics. Reproduced from ref 102. Available under a CC-BY 4.0 license. Copyright 2024 Thompson et al. (b) Left side: Effective steady-state temperature T_*exc*_ of the bright exciton as a function of the lattice temperature T. The shaded region indicates the phonon-bottleneck effect, i.e., T_*exc*_ is higher than expected from a Boltzmann distribution (black line). Right side: Experimentally obtained exciton temperatures for a pulsed (blue dots) and continous-wave (green dots) excitation. Adapted from ref 102. Available under a CC-BY 4.0 license. Copyright 2024 Thompson et al. (c) In-plane tilting (θ_*in*_), out-of-plane tilting (θ_*out*_), and variable-temperature PL spectra for (A and B) (BA)_2_PbBr_4_ and (C and D) (HIS)­PbBr_4_. Adapted from ref 106. Available under a CC-BY 3.0 license. Copyright 2017 Smith et al. (d) Normalized false-color plots of the PL from fluorinated (PEA)_2_PbI_4_ flakes with (left) and without (right) significant broad emission at room temperature. Reproduced from ref 36. Available under a CC-BY 4.0 license. Copyright 2020 Kahmann et al.

Another manifestation of strong exciton–phonon coupling concerns the remarkable diversity of emission lineshapes observed across layered perovskites. Whereas some compounds exhibit narrow excitonic emission with relatively modest Stokes shifts,^9^ others display broad sub-bandgap emission or even broadband white-light emission.^108–110^ These striking differences are frequently discussed in terms of self-trapped excitons, in which the exciton locally distorts the lattice and becomes trapped in its own deformation field.^111^ This interpretation is supported by empirical correlations between broadband emission and structural motifs expected to enhance exciton localization and exciton–phonon coupling, including large octahedral distortions in (001)-oriented Pb–Br layered perovskites^106^ (Figure 3c) and corrugated non-(001) inorganic frameworks.^108–110,112,113^ This correlation is made concrete in Figure 3c, which contrasts two Pb–Br 2D HPs: the weakly distorted (BA)_2_PbBr_4_ (BA = butylammonium), exhibiting a narrow free-exciton line, and the more strongly tilted (HIS)­PbBr_4_ (HIS = histammonium, 4-(2-ammonio­ethyl)-1*H*-imidazol-3-ium), in which a broad, strongly Stokes shifted band emerges on cooling and dominates PL at cryogenic temperatures. Across a series of (001) Pb–Br 2D perovskites, the relative weight of this broad emission scales with the out-of-plane octahedral tilt of the Pb–Br–Br bridges, providing an empirical basis for the self-trapping interpretation.^106^ More recent structural descriptors relating broadband emission to metal-cation displacement and to bond-angle variance further reinforce this correlation between ground-state lattice distortion and the propensity for self-trapping.^114,115^

At the same time, the microscopic origin of broad emission remains unresolved, and self-trapping is not the only mechanism that has been invoked. A striking counterpoint is shown in Figure 3d, which compares two flakes of fluorinated (PEA)_2_PbI_4_: nominally the same material, yet one exhibits pronounced broad emission at room temperature while the other shows almost none.^36^ Because intrinsic self-trapping should produce reproducible emission across flakes of one compounds, this variability points instead to an extrinsic, sample-specific origin. Consistent with this, both experiment and first-principles calculations have implicated permanent intrinsic defects and color centers: saturating or sublinear PL power dependence points to permanent rather than transient traps, and DFT calculations attribute the associated emission to halide-vacancy related color centers and shallow trap state.^116,117^ An impurity-induced route has also been demonstrated, in which isoelectronic doping creates local potential wells that trigger extrinsic self-trapping in a host that does not self-trap in its pure form^118^ It is therefore not clear in all cases whether the relevant mechanism is intrinsic self-trapping or instead reflects extrinsic defects, disorder, or other nonradiative pathways.^37,119–121^

A more complete description of exciton relaxation in 2D HPs may also require going beyond the discrete fine-structure levels discussed above and considering the dispersion of excitons as a function of their center-of-mass momentum **Q**. This matters because phonon-assisted relaxation, diffusion, and radiative recombination depend on how exciton population is redistributed within this dispersion. Recent theoretical work has emphasized that the exciton band structure cannot, in general, be inferred directly from the underlying electron and hole bands: exchange interactions can introduce nonanalytic features whose form depends on dimensionality and symmetry, with possible consequences for exciton dynamics, transport, and radiative lifetimes.^122^ In 2D HPs, a more complete understanding of exciton relaxation may therefore require tracking how phonons, disorder, and symmetry-breaking perturbations redistribute exciton population in momentum space and couple the optically bright **Q** = 0 manifold to finite-**Q** states.

## Unconventional Band Edges and Hybrid Excitations

Throughout this Mini-Review so far, we have assumed that the band edges of 2D HPs are derived from the inorganic sublattice, with the organic spacers acting as electronically inert dielectric barriers. In this conventional picture, the band edge dispersion is parabolic and excitonic properties follow naturally within an effective-mass framework. This picture can break down when larger π-conjugated, electro-active organic spacers are introduced. If the highest occupied (HOMO) or lowest unoccupied (LUMO) molecular orbital of the spacer is energetically aligned with the inorganic VBM or CBM, the organic and inorganic states can become electronically coupled. Because the organic states are spatially localized, and therefore give rise to flat electronic bands, their participation in low-energy excitonic transitions cannot be captured by a simple parabolic effective-mass model and can yield excited states with hybrid organic–inorganic character. Combined with the vast chemical tunability of large organic spacers, this opens up a broad design space for a subclass of 2D HPs with electronically active organic spacer molecules.^123^

The starting point for understanding this design space is the alignment of organic and inorganic frontier orbitals,^124^ which determines whether photogenerated electrons and holes localize on the inorganic or the organic sublattice, or are spatially separated between the two. Four idealized cases follow naturally (Figure 4a): In Type I_*a*_ and I_*b*_ both band edges are derived from organic or inorganic states, respectively; in Type II_*a*_ and II_*b*_ the electron and hole are spatially separated, with holes on the inorganic and electrons on the organic layer or vice versa. In principle, the organic frontier orbitals could also lie deep within the inorganic band gap, as has been observed in 3D perovskite analogues,^125^ although such configurations remain largely unexplored in 2D HPs. Type I_*b*_, with organic states far from the band edges, has been by far the most extensively studied, and is the “conventional” case discussed in much of this Mini-Review.

**Figure 4:**
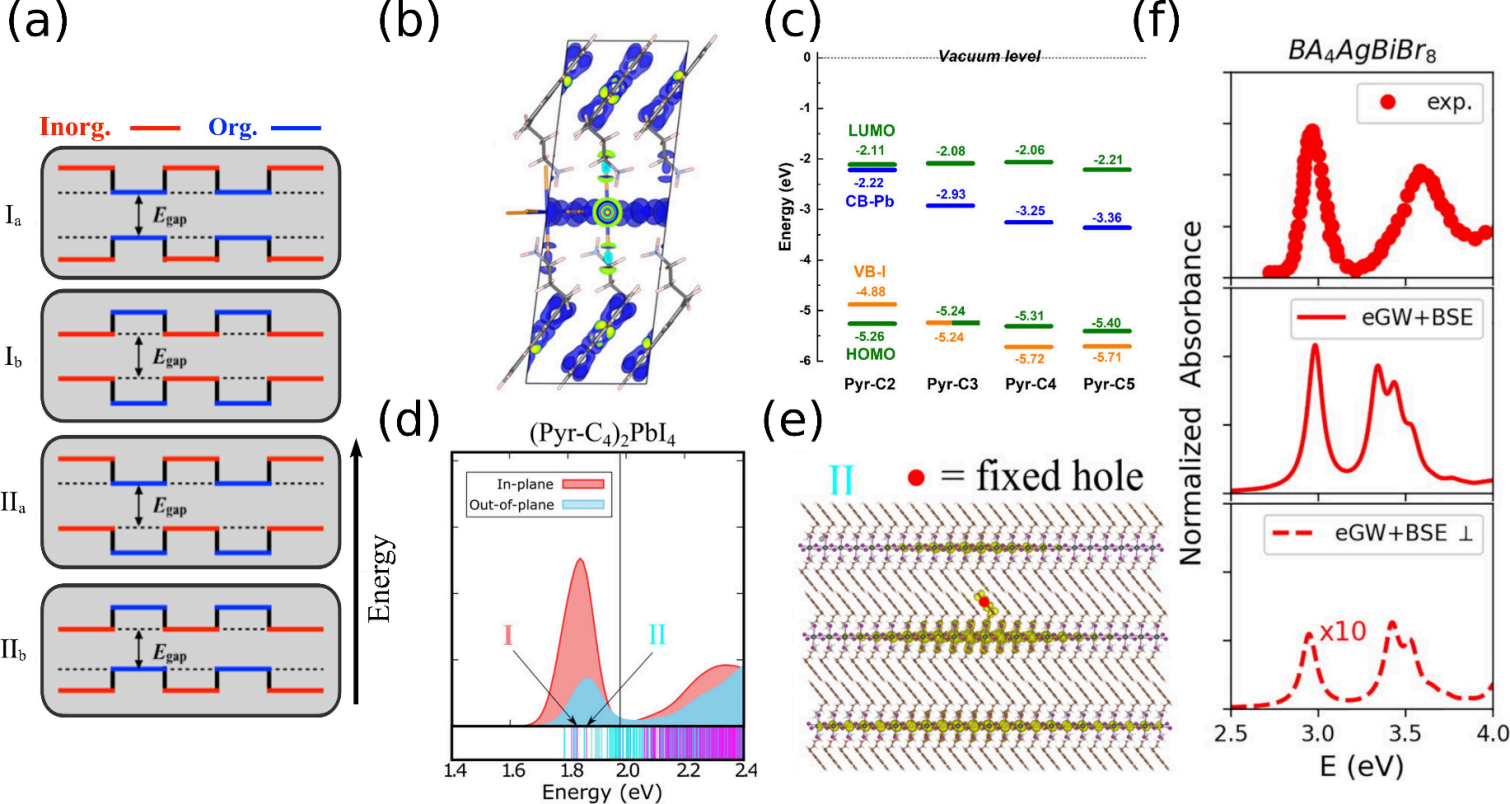
(a) Possible energy level schemes (I_*a*_, I_*b*_, II_*a*_, II_*b*_) for the alternating organic–inorganic perovskite structure, with the overall band gap indicated by arrows and dashed lines. Adapted from ref 124. Copyright 2018 American Physical Society. (b) Partial charge density distribution of the electronic states in (Pyr-C_3_)_2_PbI_4_, calculated using DFT-HSE. Reproduced from ref 126. Copyright 2024 American Chemical Society. (c) Sketch of the DFT-HSE energy-level alignments between the Pyr-C_2_/C_4_ spacers and the perovskite framework in the (Pyr-C_2_)_2_PbI_4_ and (Pyr-C_4_)_2_PbI_4_ monolayers. The organic molecular orbital levels and perovskite Pb and I frontier levels are color-coded in green, blue, and orange, respectively. Reproduced from ref 126. (d) *G*_0_ *W*_0_+BSE calculated absorption plot and spectrum for (Pyr-C_4_)_2_PbI_4_. The black line indicates the *G*_0_ *W*_0_@PBE+SOC band gap. The light-blue component indicates the out-of-plane absorption, and the red component indicates the in-plane absorption. The bars below the spectra indicate excitation energies, colored magenta for inorganic–inorganic character or cyan for organic–inorganic character. The brightest in-plane and out-of-plane excitons are indicated with I and II, respectively. Reproduced from ref 101. (e) Exciton wave function density of exciton II for (Pyr-C_4_)_2_PbI_4_ with the hole fixed on the pyrene molecule. Reproduced from ref 101. Copyright 2025 American Chemical Society. (f) Top panel shows experimental absorption spectrum from ref 127 for (BA)_4_AgBiBr_8_. The middle and bottom panels show calculated absorption spectra for unpolarized light (solid line) and light polarized perpendicular to the plane of the inorganic sheets (dashed line). Reproduced from ref 128. Copyright 2021 American Institute of Physics.

The earliest 2D HPs falling outside this regime were reported in 1999, with quaterthiophene-based systems by Mitzi et al.,^129^ and pyrene-based systems by Braun et al.,^130^ both incorporating dye molecules with HOMO–LUMO gaps smaller than the inorganic band gap. Modern interest in Type II alignments has been driven by the prospect of enhanced out-of-plane carrier transport and interlayer energy transfer,^124,131^ of particular interest for solar-cell applications.^132^ Building on this motivation, Ni et al. investigated electronically active, pyrene-based spacers, where π-conjugated stacking of the pyrene cores (Figure 4b) places the HOMO energy close to the inorganic VBM.^126^ This is shown in Figure 4c: In (Pyr-C_3_)_2_PbI_4_, the valence (hole) charge density is no longer confined to the inorganic slab but spreads across both the π-stacked pyrene cores and the PbI_4_ framework. Using first-principles DFT, they showed that the HOMO position can be tuned by varying the alkylammonium chain length attached to the pyrene core (Figure 4c), and predicted a Type I_*b*_ to Type II_*b*_ crossover at three carbon atoms (Pyr-C_3_), although only Pyr-C_2_ and Pyr-C_4_ had been successfully synthesized at the time. Such alignment near the crossover is expected to increase out-of-plane hole mobility and to enable interlayer hybrid excitons.^96,133,134^

Boeije et al. revisited this picture for (Pyr-C_4_)_2_PbI_4_ using a combination of first-principles *GW*+BSE calculations, single-crystal optical spectroscopy, and transient optical microscopy,^101^ concluding that the crossover from Type I_*b*_ to Type II_*b*_ in fact occurs for (Pyr-C_4_), where the pyrene HOMO and VBM lie at nearly the same energy. They further showed that energetic alignment alone is not sufficient: hybridization between organic and inorganic states additionally requires a molecular orientation that provides adequate π-orbital overlap with the electronic state derived from the inorganic framework. Consistent with this picture, (3T)_2_PbI_4_ (T = thio­phenyl­ethyl­ammon­ium), a Type II_*b*_ system with HOMO and VBM levels comparable to those of (Pyr-C_4_)_2_PbI_4_, shows qualitatively different excitonic emission that suggests a much lower degree of organic–inorganic hybridization,^135^ reinforcing the conclusion that band alignment alone is an insufficient descriptor. Where the geometric criterion is met, by contrast, hybridization is captured at the independent-particle level and propagates to the excitonic level through the mixing of organic–inorganic and inorganic–inorganic transitions in the BSE solution (Figure 4d). This is shown in Figure 4e, which plots the electron density of the brightest out-of-plane exciton with the hole pinned to the pyrene unit: rather than remaining within a single inorganic layer, the electron leaks across adjacent PbI_4_ sheets, visualizing an interlayer-delocalized, organic–inorganic hybrid exciton. This hybrid character strongly enhances the optical activity and spatial delocalization of out-of-plane excitons and can lead to enhanced out-of-plane exciton mobility.^101^ Beyond this work, studies of hybrid excitons in these types of systems remain sparse: Forde et al. reported a charge-transfer state in NEA-based (naphthylethylammonium) systems without organic–inorganic hybridization at the DFT level,^136^ while Liu et al. using quasiparticle self-consistent *GW* with vertex corrections recovered a markedly different band alignment for the same system,^86^ highlighting the strong sensitivity of predicted band alignments to both structural details and level of theory.

Finally, we note that a complex band-edge electronic structure in 2D HPs is not restricted to cases with electronically active organic spacers. A qualitatively different route arises through variation of the metal-cation sublattice, most prominently in halide double perovskites, where alternating *B* and *B*′ cations generate a much broader range of band gaps, orbital characters, and band-edge symmetries than in simple Pb-based compounds.^137,138^ In such systems, the frontier states may no longer be well described by the conventional Pb-*p* conduction band and Pb-*s*/halide-*p* valence band picture, and the resulting dispersion can be indirect, strongly anisotropic, unusually flat, or otherwise poorly captured by a simple isotropic parabolic effective-mass model.^139^

Their 2D congeners typically inherit important aspects of the band-edge character of the 3D parent compounds, but dimensional reduction can further modify the band-edge symmetry and dispersion.^127,140,141^ This is particularly evident in Ag-containing 2D halide-double perovskites, where the directional Ag *d* orbitals contribute significantly to the valence-band edge and drive an indirect-to-direct band gap transition in (BA)_4_AgBiBr_8_ and related compounds.^141^ More broadly, the double perovskite stoichiometry also opens a route toward magnetic 2D HPs through incorporation of open-shell metal cations, as already demonstrated in Ag/Fe-based 2D HPs,^142^ thereby extending this family toward magnetic and magneto-optical functionality. Although excitonic fine structure and related excited-state properties remain much less explored in this family than in Pb-based layered perovskites, first-principles studies point to strongly bound and localized excitons,^143^ pronounced anisotropy^128^ (Figure 4f), and unusual exciton scaling behavior linked to structural distortions driven by Ag *d*-orbital chemistry.^144^ We therefore mention 2D halide double perovskites here as a second important class of layered materials in which the simple band-edge picture can break down, even in the absence of direct organic-state participation.

## Outlook

As this Mini-Review hopefully makes clear, the excitonic physics of 2D HPs is far richer than a simple picture of strongly bound but otherwise conventional quantum-well excitons may suggest. Considerable progress has been made in establishing the basic ingredients that govern their low-energy excited states, including exchange interactions, spin–orbit coupling, dielectric confinement, structural symmetry breaking, and coupling to phonons. At the same time, several central questions remain open. A first is how to connect the now well-established idealized fine-structure picture to the full spectral complexity observed in specific compounds, where inequivalent layers, local symmetry breaking, exciton–polaron effects, defects, and charged excitonic complexes may all contribute. A second is how to move beyond static descriptions of excitons toward a microscopic understanding of their dynamical behavior, including phonon dressing, bright-dark relaxation, finite-**Q** population redistribution, and the origin of broad versus narrow emission. A third is how the vast existing design space of layered perovskites can be exploited to uncover qualitatively new excitonic phenomena and functionalities. Beyond conventional Pb-based compounds, this space already includes chiral and electronically active spacers, alternative metal and halide chemistries, layered halide double perovskites, corrugated and otherwise non-(001)-oriented frameworks, and the emerging class of magnetic 2D HPs. The challenge is therefore to understand which aspects of this structural and chemical diversity give rise to genuinely new excited-state physics, and which can be harnessed for functionalities such as polarized and chiral emission, selective spin transport, or improved luminescence.

Although our focus has been on the fundamental excitonic physics of 2D HPs rather than on devices, several of the properties discussed here affect macroscopic optoelectronic performance. For light emission, the ordering of the bright and dark states and the size of the bright-dark splitting affect radiative efficiency, since a low-lying dark state can trap population and quench luminescence; the phonon-bottleneck effect that holds bright-state populations above their thermal-equilibrium value is one mechanism by which this limitation can be circumvented.^102^ The orientation of the excitonic transition dipoles is similarly relevant, as in-plane versus out-of-plane dipole character governs the outcoupling of light from thin-film emitters, while the strong exciton–phonon coupling responsible for broad, Stokes-shifted emission underlies the use of some layered perovskites as single-component white-light phosphors.^109^ Chiral and spin-dependent optical responses translate, in principle, into circularly polarized emission and spin-selective transport of interest for chiral photonics and spintronics.^81–86^ For photovoltaics, by contrast, the large exciton binding energies that make these materials such a rich excitonic platform are in tension with the efficient free-carrier generation required for charge extraction, which is one reason why 2D HPs are most often employed in higher-*n* quasi-2D form or as stabilizing layers atop 3D absorbers. Electroactive-spacer strategies which promote out-of-plane charge separation and transport through Type II band alignments and interlayer hybrid excitons offer a route to improve device-relevant transport. These connections are largely indirect and remain to be quantified systematically, but they indicate that the excitonic fine structure, its dynamics, and its symmetry are not only of fundamental interest but also important for the optoelectronic function of this materials class.

Addressing open questions in this field requires a close interplay between targeted and well-controlled synthesis, spectroscopy and other experimental characterization techniques, as well as theoretical and computational approaches that can bridge several levels of description. First-principles numerical simulation methods are essential for establishing structure–property relationships on a material-specific footing. However, many of the most interesting open problems in this field demand theoretical and computational frameworks that combine electronic structure, lattice dynamics, and excitonic correlations more explicitly than is currently routine. In this sense, 2D HPs are not only a highly tunable family of excitonic materials, but also a demanding test bed for first-principles atomistic frameworks of excited states in soft quantum materials. If these challenges can be met, 2D HPs may become a uniquely versatile platform for spin-optical functionality, chiral and polarized light emission, and other excitonic optoelectronic and quantum phenomena in low dimensions.
